# Copper Toxicity Is Not Just Oxidative Damage: Zinc Systems and Insight from Wilson Disease

**DOI:** 10.3390/biomedicines9030316

**Published:** 2021-03-20

**Authors:** R. G. Barber, Zoey A. Grenier, Jason L. Burkhead

**Affiliations:** Department of Biological Sciences, University of Alaska Anchorage, 3211 Providence Dr., Anchorage, AK 99058, USA; rgbarber@alaska.edu (R.G.B.); zgrenier@alaska.edu (Z.A.G.)

**Keywords:** copper, zinc, Wilson Disease, copper toxicity, oxidative stress

## Abstract

Essential metals such as copper (Cu) and zinc (Zn) are important cofactors in diverse cellular processes, while metal imbalance may impact or be altered by disease state. Cu is essential for aerobic life with significant functions in oxidation-reduction catalysis. This redox reactivity requires precise intracellular handling and molecular-to-organismal levels of homeostatic control. As the central organ of Cu homeostasis in vertebrates, the liver has long been associated with Cu storage disorders including Wilson Disease (WD) (heritable human Cu toxicosis), Idiopathic Copper Toxicosis and Endemic Tyrolean Infantile Cirrhosis. Cu imbalance is also associated with chronic liver diseases that arise from hepatitis viral infection or other liver injury. The labile redox characteristic of Cu is often discussed as a primary mechanism of Cu toxicity. However, work emerging largely from the study of WD models suggests that Cu toxicity may have specific biochemical consequences that are not directly attributable to redox activity. This work reviews Cu toxicity with a focus on the liver and proposes that Cu accumulation specifically impacts Zn-dependent processes. The prospect that Cu toxicity has specific biochemical impacts that are not entirely attributable to redox may promote further inquiry into Cu toxicity in WD and other Cu-associated disorders.

## 1. Introduction

### 1.1. Copper

Cu (atomic number 29, average molecular weight 63.55) is an essential micronutrient with electronic configuration [Ar] 3d^10^4s^1^ and placement in group XI, period IV of the periodic table. This configuration is important to explain the reactivity of Cu and its enzymatic utility through labile oxidation and reduction between Cu(I) and Cu(II) under physiological conditions. However, unbound or “free” Cu ions are not generally encountered in cells in either valence state (for a detailed discussion of the chemical speciation of Cu in cells, see Fahrni [1]). Cu is essential for many physiological processes including iron homeostasis [2], angiogenesis [3], neurotransmitter biosynthesis [4], immune function (including neutrophil activation and macrophage function) [5] and energy metabolism [6].

### 1.2. Normal Copper Metabolism and Its Relationship with Liver

The transition metal Cu is an essential trace element that must be acquired from the diet. Its high redox potential readily permits electron transfers to oxygen [7]. This property allows Cu to serve as an indispensable cofactor for enzyme function, but also requires that Cu be sequestered to avoid the generation of reactive oxygen species. Given its unique anatomical location and microcirculatory anatomy, the liver is a master regulator of nutrient metabolism, including Cu. The absorption, uptake, export, and transport of Cu are tightly regulated because both too much and too little Cu are associated with oxidative cell damage, compromised immune function and organ dysfunction. In liver parenchymal cells, Cu is locally utilized for cellular processes including mitochondrial respiration and free radical detoxification, while it is also actively transported into the trans-Golgi network via ATP7B transporters [8]. Excess Cu can be sequestered by metallothionein (MT) proteins, as well as exported via ATP7B, which traffics to endo- or lysosome-derived compartments and the apical (bile canalicular) membrane [9,10]. Alternately, Cu exits hepatocytes via the basolateral membrane as a cofactor of the ferroxidase ceruloplasmin [11]. Blood circulating Cu can be acquired by other tissues including the brain, kidney, heart, connective tissue, and pancreas [12]. Although most whole-body Cu regulation is attributed to ATP7B, the Cu exporter ATP7A has central roles in intestinal Cu acquisition and mobilization across the blood–brain barrier [13]. ATP7A may also function to mobilize Cu from the liver to the bloodstream across the hepatocyte basolateral membrane in order to serve specific organ systems [14]. The cellular localization of Cu in the liver may be important in Cu homeostasis. For example, high Cu localization to lysosomes is found in the neonatal cholestatic liver [15]. Additionally, high cytosolic Cu in cholestatic hepatocytes is similar to the diffuse localization of Cu in the early stages of Wilson Disease (WD) [15]. In summary, Cu metabolism and liver function are closely intertwined.

### 1.3. Copper Toxicity

Cu toxicity in humans can be a result of mutations in genes encoding Cu handling machinery, environmental exposure (typically via consumption) or a combination of causes. Elevated Cu levels are also associated with liver injury due to viral infection or cholestatic disease. Cu is expected to promote oxidative damage; however, it is not clear that this is the primary cause of toxicity. Studies in animal models suggest that in response to excess Cu, cell redox buffering may mitigate acute oxidative stress, yet fail to compensate for ancillary biochemical impacts such as altered lipid metabolism, impaired transcriptional activation or mitochondrial fragmentation [16,17,18,19]. Bearing these observations in mind, the objectives of this review are as follows: (1) Provide an overview of Cu toxicity in humans and in animal models, (2) Examine evidence that Cu toxicity has specific molecular consequences independent of Cu-induced oxidative damage, and (3) Propose a model where Cu selectively impacts Zn dependent molecular machinery including Zn containing transcriptional activators and enzymes with Zn cofactors.

## 2. Copper Excess and Consequences of Copper Toxicity in Humans and Animal Models

### 2.1. Copper in Liver Disease: Viral Hepatitis

Liver injury or altered function due to viral infection may impact systemic or organ-level Cu balance. Elevated serum Cu (along with iron) has been reported in patients with hepatitis C virus (HCV), with significant increases in cirrhotic patients [20,21,22]. In patients with anti-HCV antibodies, increases in essential transition metals were also positively correlated with markers of oxidative stress [21]. Increases in serum transition metals have also been studied in hepatitis B virus (HBV) cases at various stages of disease, finding that HBV patients with hepatocellular carcinoma (HCC) had increased serum Cu but decreased Zn, Fe and Se levels compared to a control group [23]. It is not yet clear if viral-induced increases in bodily Cu contribute to liver injury or pathology, though this is an area currently under study.

A study in female patients reported increased serum and urine Cu and Fe in viral hepatitis A, B, C, D and E, while the strongest metal associations with hepatitis viruses were significant Cu increases in the scalp hair of viral hepatitis A, B and C patients [24]. These studies suggest that viral liver injury can cause changes in systemic Cu balance, although increased serum or plasma Cu may be due to acute infection and ceruloplasmin production [25]. A study of liver disease progression in chronic HCV found that liver Cu content increased with the progression of fibrosis and was positively correlated with bilirubin [26]. This work also reported a negative correlation between Cu and albumin with no fibrosis grade-associated changes in Fe or Zn. A histological staining analysis in chronic active viral hepatitis reported that Cu and Fe deposition was associated with lower proliferative capability, suggesting Cu deposition zones are associated with necrosis in these cases [27]. High Cu in HCC in chronic hepatitis or cirrhosis caused by HCV indicated increased Cu in liver parenchymal tissue with HCC as opposed to tissue without HCC, while a similar increase in Fe levels was not observed [28]. These studies link Cu accumulation with viral liver injury, particularly implicating hepatic Cu in HCV progression. However, a mechanism of action where Cu promotes liver injury in these pathologies is not readily apparent.

### 2.2. Copper in Liver Disease: Cholestatic Liver Disorders

Hepatic Cu accumulation can result from cholestatic liver disorders, most notably in primary biliary cirrhosis (PBC), where it appears that normal levels of biliary Cu excretion are not sufficient to remove Cu accumulated in the liver [29,30]. Treatment with D-penicillamine (D-PEN) has been tested in PBC as a Cu depletion strategy; however, D-PEN was not found to be effective in reducing accumulated hepatic Cu or mortality risk [29,31].

### 2.3. Copper Toxicity from Excess Consumption

Acute Cu toxicity from consumption is not well understood in humans. The literature includes a number of case reports documenting acute toxicity from the ingestion of Cu-laden beverages with many cases exceeding 20 g. A series of cases reporting chronic Cu poisoning due to tap water contamination in Germany revealed largely gastrointestinal symptoms and excess non-ceruloplasmin Cu in blood and urine [32,33]. Cases of intentional acute Cu ingestion are often intentional self-harm, can be fatal, and are characterized by damage to erythrocytes, the liver and kidneys (two case reports with literature reviews are illustrative: [34,35]). Molecular mechanisms of such acute toxicity are not characterized.

Chronic Cu toxicity through excess consumption has been studied in animal models. These studies examine consequences of chronic sublethal Cu “loading” including a series of reports in the “Cu-loaded rat” fed 1 g/kg Cu for 16 weeks. This experimental series reported kidney Cu sequestration in MT-containing granules and association with higher molecular weight proteins in the liver [36]. Increases in circulating and urinary Cu and MT were reported, suggesting a non-biliary route of excretion may be activated by excess Cu [37]. Further study reported Cu in the nucleus, nucleolus and lysosomes of hepatocytes, indicating a partitioning system in cells [38].

Work in a diabetic vs. non-diabetic male rat model treated with a 30-day regime of 10 or 60 mg/kg Cu/day via gastric tube reported adverse Cu-induced changes to serum lipid profiles and decreased antioxidant enzyme activity with greater susceptibility in diabetic rats, indicating that Cu toxicity may be augmented by diabetes [39]. Similarly, mice fed a range of 0–16 mg/kg/day Cu via gavage exhibited inflammatory responses and liver injury in response to Cu administered above 4 mg/kg/day [40]. Further, an important study in rats quantified malondialdehyde (MDA) by the thiobarbituric acid reaction as a readout of lipid peroxidation in response to long-term dietary Cu [41]. Although histological lesions were induced by Cu consistent with dose-response (six diet groups from 4.8–766 µg/g Cu), there were no significant differences in MDA levels between groups.

In another long-term dietary Cu loading experiment, *Cebus capucinus* monkeys were fed a high Cu diet that increased from an initial 5 mg/kg/day Cu (as Cu gluconate) to 7.5 mg/kg/day over two months. The feeding regime was maintained for three years, resulting in hepatic Cu increases of approximately 5-fold (~15 vs. ~75 µg/g dry weight) in Cu-treated animals (>250 µg/g hepatic Cu is characteristic of WD [42]). This treatment promoted a transcriptional response consistent with inflammation and proliferative gene expression. However, there was no apparent blood chemistry, clinical or histological indication of damage [43]. The Cu-loading experiment in *C. capucinus* was followed up in the same animals by a one-week break from Cu treatment and subsequent challenge with an acute toxic dose of acetaminophen [44]. Although liver pathology was not apparent, the Cu-treated animals exhibited an attenuated transcriptional response to acetaminophen as well as decreased liver injury compared to non-Cu treated animals as measured by serum alanine transaminase (ALT). This observation suggests broad Cu tolerance and capacity to prevent toxicity in mammals via both adaptive and homeostatic responses.

### 2.4. Human Copper Toxicosis—Wilson Disease

Wilson Disease is the most well-documented disorder of Cu toxicity and is an autosomal recessive disorder of Cu metabolism with an estimated global allele frequency of 1:90 [45]. This rare disease is caused by a mutation in the *ATP7B* gene [46] that is characterized by excessive Cu accumulation, primarily in the liver, but also in other tissues including the brain [47]. The disorder is potentially lethal if untreated. WD can lead to diverse clinical manifestations such as hepatitis, cirrhosis of the liver, and liver failure, as well as neurological symptoms including tremors, dystonia, and psychological conditions [48,49,50]. Symptom onset in WD patients typically begins between the ages of 3 and 40, while initial observations often include neurological or behavioral symptoms akin to Parkinson’s Disease in addition to symptoms of liver disease. Diagnosis is usually established after the evaluation of serum ceruloplasmin (CP, which is typically below the reference range in WD), 24hr urinary Cu excretion and a slit lamp examination for the presence of Kayser–Fleischer rings (indicative of corneal Cu deposition) [42].

The neuropathology for WD is well defined and deserves specific attention. However, less is understood about neurologic compared to hepatic WD as well as the relationship between hepatic and brain Cu toxicity. Cu accumulates nonspecifically in the WD brain at concentrations up to 10 times higher than normal [51]. Astrocytes are the primary regulators of brain Cu loading. In WD, they grow in number and in size as they store large quantities of Cu locally, as well as produce abnormal astrocytic cells known as Alzheimer type I glia and Opalski cells. This process impairs normal astrocyte function in the brain, which may contribute to the neuropathology of WD through damage to neurons and oligodendrocytes [52]. Poujois et al. (2017) provide an up-to-date review of WD brain pathology in patients with neurologic symptoms, including the lack of correlation between cerebral Cu and symptom severity and the implication that WD brain pathology may be more complex than direct Cu toxicity [53]. Furthermore, it is notable that WD is one of the few neurodegenerative disorders that can be successfully treated with pharmacological therapies as found in multiple long term followup studies [54,55,56].

Wilson Disease shares many symptoms with other neurological diseases such as Alzheimer’s and Parkinson’s—diseases that are also characterized by Cu accumulation in the brain [57]. It is possible that study in WD may illuminate etiologies in these more common pathologies.

Since the underlying disease mechanisms are not fully understood, there are resultant limitations on our understanding of WD treatment and its metabolic consequences. Therapies for WD include the consumption of a low-Cu diet, treatment with de-coppering agents such as D-PEN, trientine, tetrathiomolybdate, and Zn supplementation. All of these treatments focus on reducing the overall Cu burden in the body. Poor response to these therapies necessitates liver transplant. While these therapies may reduce hepatic Cu accumulation, they do not always ameliorate and can even induce neurological symptoms [58]. For example, tetrathiomolybdate treatment was reported to have negative neurological impacts in 9% of patients in one study [59]. It is possible that these responses may be due to a temporary transference of Cu from the liver to the circulation, worsening the overall Cu stress. Alternatively, WD treatment might negatively impact brain homeostasis of Cu or other transition metals such as Zn.

### 2.5. Non-Wilson Copper Toxicosis

Non-Wilson Cu toxicosis is also recognized in several other liver disorders including Idiopathic Copper Toxicosis (ICT), Endemic Tyrolean Infantile Cirrhosis (ETIC), and Indian Childhood Cirrhosis (ICC), which can all manifest as cirrhosis with Cu accumulation. These diseases are characterized by liver Cu accumulation without *ATP7B* mutation, though they do appear to have both a genetic and an environmental (i.e., high Cu in the diet) component [60,61,62]. These non-WD disorders associated with hepatic Cu overload have varied phenotypes including onset in infancy as opposed to later in life as in WD [62]. The divergence of ICT onset as well as pathology from that of WD is proposed as a synergy between an unknown genetic factor and high dietary Cu [63,64]. Similarly, ETIC is another early-onset Cu disorder that can lead to fatal hepatic insufficiency and cirrhosis. Evidence supports familial clustering in ETIC [65], with clinical profiles divergent from WD including: CP within reference range, and high ALT, aspartate transaminase (AST), and gamma-glutamyltransferase (GGT). Both ICT and ICC also diverge from WD in that they are both characterized by elevated serum Cu: individuals with ICC have increased serum Cu compared to controls and family members [66], while ICT is described with serum Cu above the reference range [65]. Importantly, ICC is strongly associated with high Cu consumption in the diet and is thus considered treatable [67,68].

Domesticated animals are also observed with Cu toxicosis. The Bedlington Terrier dog was initially proposed as a canine model for WD over 40 years ago with hepatic Cu concentrations ranging up to nearly 10,000 µg/g (dry weight) in liver tissue [69,70]. This Cu accumulation was predominantly localized to lysosomes, while the causative mutation is not in *ATP7B* but was rather identified in a gene initially called Murr1 (later named COMMD1) [71]. Other dog breeds have been found with elevated hepatic Cu, including Labrador Retrievers homozygous for *ATP7B* mutation [72,73]. Interestingly, some Labrador Retrievers also have an ATP7A variant that is protective through apparent delayed Cu accumulation [73]. The Labrador Retriever was reported in 2016 as the first natural, non-rodent variant WD model, though the mechanistic characterization of Cu toxicity has not been extensively explored.

It is clear from both WD and non-WD Cu toxicosis that Cu accumulation is hepatotoxic as determined by clinical values indicative of liver injury. Underlying these studies as well as Cu-loading work in animal models is the understanding that the liver and other tissues have homeostatic and adaptive mechanisms to mitigate the stress of excess Cu. The variability in the age of onset for WD and other disorders of Cu metabolism also suggests Cu toxicity mechanisms that are an accumulation of damage at the molecular level. Therapy for Cu accumulation disorders focuses on relieving the Cu burden, as noted for WD. These therapies may also offer insight into molecular mechanisms of Cu toxicity.

### 2.6. Zinc as a Therapy for Wilson Disease—Competition with Copper and Negative Copper Balance

Oral Zn treatment for WD was first proposed by Schwudick in a thesis [74] and later elaborated by Hoogenraad, illustrating the inhibition of Cu absorption in the intestine [75]. Current practices mirror this treatment [42]. We now know that MT transcription is regulated by cellular Zn levels and that MT preferentially binds to Cu [76,77]. Thereby, in the presence of high Zn concentration, MT transcription is upregulated and preferentially binds and retains Cu in enterocytes. Thus, the current conceptual model indicates that the induction of MT in the intestine sequesters excess Cu in the epithelial cells, preventing transfer into the bloodstream and thus creating a negative Cu balance [78].

Zn is currently a first-line treatment in presymptomatic cases of WD as well as long-term treatment of recovering symptomatic WD patients. Oral Zn is supported by long-term clinical studies that report improved hepatic, neurological and excretory symptoms. Studies that include both Zn and chelator pharmaceuticals are included in Table 1. These findings indicate positive outcomes in a number of trials and retrospective studies for both Zn and chelator treatments.

Zn is well tolerated among WD patients in long-term studies, particularly when compared to D-PEN, and has relatively few side effects, chiefly gastric irritation. Zn acetate is approved as a WD maintenance therapy in the United States and Europe, while other salts including Zn gluconate, Zn sulfate and Zn picolinate are also available as nutritional supplements. A single-center retrospective study reported similar therapeutic results with different salts [79]. Treatment with Zn appears to improve neurological WD symptoms over long periods of time (>1 year) [54,55,56]. Other responses to oral Zn include decreases in both non-CP bound plasma Cu and 24-h urinary Cu excretion with improved markers of hepatic enzyme function [55,80]. Additionally, there is conflicting evidence to suggest that Zn treatment can reduce hepatic Cu load over time [56,81]; however, in cases of hepatic failure, Cu chelators such as D-PEN are more effective at preventing hepatic deterioration [82,83]. Studies on both chelators and Zn reveal largely similar patient outcomes in WD, with improved metrics such as those stated above. Specifically, in cases of extreme Cu load and symptoms, Zn has a higher rate of treatment failure [83,84]. For example, in a 2011 review by Weiss [85], Zn had a significantly higher rate of treatment failure when compared with chelators. Additionally, in long term studies, chelators are less likely to be well-tolerated by patients, while switching treatment to oral Zn typically has a greater rate of patient compliance [84,86,87]. Furthermore, some patients exhibit deteriorating neurological symptoms under treatment with D-PEN, some of whom improved only after treatment was switched over to Zn [84,88,89]. This is thought to be due to an increased mobilization of free Cu that is associated specifically with D-PEN [90,91].

Notably for this review, the therapeutic effects of Zn supplementation, as well as its limitations, provide an initial clue into molecular mechanisms of Cu toxicity. The induction of MTs by Zn suggests that Cu excess may interact with or influence cellular Zn handling machinery and that this interaction may extend beyond the intestinal epithelium targeted by oral Zn therapy. The discrete response of MTs to both Cu loading and Zn therapy as discussed above suggests that Cu excess might selectively target other Zn-associated systems. The global therapeutic effect of oral Zn treatment in WD is illustrative of this concept.

## 3. Specific Targets of Copper Toxicity in Wilson Disease

A major challenge in understanding how Cu toxicity causes WD pathology, as well as that of other Cu accumulation disorders, has been the identification of functional links between global Cu accumulation and subcellular damage. Specifically, the mechanism by which Cu accumulation affects cellular function is not well understood. A general mechanism of oxidative stress is often cited [99] and is supported by evidence in advanced stages of Wilson-associated liver disease or in the early-onset model Long-Evans Cinnamon (LEC) rat [100,101,102]. However, the variability of symptoms and onset, even among monozygotic twins [103], suggests that specific biochemical pathways or mechanisms may be impacted by Cu, particularly in WD. Thus, the search for impacts on cellular and molecular processes in WD has gained recent attention and has illuminated both disease mechanisms and potential therapeutic targets.

### 3.1. Metabolic Consequences of Copper Toxicity in Wilson Disease Models

Animal models of WD, including the spontaneous *Atp7b* mutant toxic milk (*tx*) mouse [104,105], *Atp7b^-/-^* mouse [106], and LEC rat [107], have provided controlled animal model data illustrating the mechanisms of Cu toxicity. These models recapitulate many hepatic symptoms of Wilson Disease but have not been extensively characterized for neurological phenotypes (summarized in a prior review [108]). The initial characterization of these rodent models focused on metal accumulation and metal-associated molecules such as MTs [109,110]. Impacts on mitochondria have been reported in mice, including oxidative stress and cardiolipin fragmentation [111], while autophagy and mitophagy pathways have been shown in the LPP rat (derived from the LEC rat) [112]. Characterization of the *Atp7b^-/-^* mouse [16,113], as well as the LEC rat and WD patients [19], indicated that direct oxidative stress from Cu is largely buffered in early disease stages, whereby oxidative damage results from secondary effects such as those in mitochondria [19]. This Cu-induced oxidative and apoptotic stress was also studied in a cell culture model where both increased reactive oxygen species and antioxidant mechanisms are upregulated with Cu challenge [114]. These types of cell and systemic stresses are expected to be broadly damaging to cell health as radical toxicity is untargeted.

An alternative route to understanding Cu toxicity, starting with WD, implements systems biology or data-driven approaches. Microarray-based transcriptomics identified downregulation in cholesterol synthesis genes in the *Atp7b^-/-^* mouse liver that suggested Cu toxicity indeed impacts specific physiological processes [16]. The synthesis of Atp7b^-/-^ mouse and LEC rat liver transcriptomics studies revealed that gene expression pathways regulated by nuclear receptors (NRs) are significantly represented, specifically in lipid metabolism regulation by Liver-X-Receptor/Retinoid-X-Receptor/Farnesoid-X-Receptor (LXR, RXR, FXR, respectively) [108]. Both models also increased proliferative gene expression while the LEC rat, studied at a later disease stage, also increased inflammatory gene expression. Subsequent untargeted proteomics study revealed decreased abundance of the FXR protein (also known as NR1H4) in six-weeks-old *Atp7b*^-/-^ mice, linking suppressed gene expression to decreased abundance of a specific transcriptional activator [115]. Transcriptional pathways regulated by NRs are of particular interest as they are consistent with decreased cholesterol levels and biosynthetic gene expression in animal models and WD patients [16,116]. More recent work found that NRs, specifically LXR, were potential therapeutic targets in WD [117] and are indeed specifically inhibited from DNA binding in vitro by excess Cu [18]. This work also found that NR activity in the *Atp7b*^-/-^ mouse is improved by dietary Zn supplementation, and verified that WD patients with high hepatic Cu have gene expression changes consistent with metabolic NR disruption [18].

### 3.2. Zinc Systems in Wilson Disease: Transcriptional Regulation and Nuclear Receptors

The results of the above-mentioned systems biology work and follow-up on NRs indicated that certain NRs might be specifically targeted by Cu excess in cells. This observation was also confirmed by treating cultured hepatoma cells with Cu and measuring either NR activity or abundance [18,118]. NRs are ligand-activated transcriptional activators with Zn finger DNA binding domains [119]. The NR Zn finger represents a distinct class, in that the DNA binding domain Zn ion is coordinated by four sulfhydryl groups provided by two C-X-X-C motifs. Other Zn finger domains coordinate Zn with a combination of two or three cysteine and one or two histidine motifs (e.g., 2His-2Cys or 3Cys-1His). Curiously, a similar C-X-X-C motif is highly conserved and provides specific linear Cu(I) coordination in Cu handling proteins Atox1 and the N-terminal Cu-binding domains of ATP7A and ATP7B [120,121]. This coordination suggests that NRs may be susceptible to the presence of excess intracellular Cu due to favorable Cu(I) binding motifs. This observation leads to the initial hypothesis that Zn-containing proteins or processes may be specifically impacted by excess Cu in WD.

A recent study constructed a transcriptional network comparing *Atp7b*^-/-^ and wild type mice, including up- and down-regulated genes. This network indicated NR involvement in a global network that contained 231 transcript and 15 transcriptional activator nodes [122]. Four NR clusters were identified with NR3C, NR4A, HNF, PPAR, RORC, and RAR families of global regulators connecting 50% of the genes contained in the network. These NRs are involved in the regulation of a wide range of functions, including cellular differentiation, development, and carbohydrate, lipid, and protein metabolism. Four additional transcriptional activator clusters included non-NR Zn finger transcription factors with the ZBTB and ZFP family proteins indicated as the most connected regulators in the network. This network derived from empirical data suggests a global targeting of Zn-finger transcriptional activators as a result of Cu excess. Therefore, much of the Cu toxicity observed in WD may be due to the disruption of numerous Zn-dependent and Zn-responsive proteins, including transcription factors. The transcriptional network also included metal-responsive transcription factor-1 (MTF-1) as a specific node. Zn is a direct activator of MTF-1, with recent data indicating MTF-1 functions as a Zn sensor [76]. MTF-1 activates the transcription of metal-binding metallotioneins (MTs) by binding to metal-responsive elements (MREs). MTs perform several functions including the modulation of intracellular essential metal homeostasis and distribution, as well as protection against the accumulation of excessive amounts of both harmful and essential metals [123]. They accomplish these functions through the binding of metals within two cysteine-rich protein domains to create metal-thiolate clusters. Considered with the established therapeutic benefit of Zn in WD, this analysis suggests a selective impact on Zn-specific machinery in cells that feature NRs and MTs but may also include other Zn-dependent proteins.

### 3.3. Zinc

In proposing that Zn machinery may be selectively targeted by excess cellular Cu, it is important to emphasize the physiological importance of Zn and its basic homeostasis. Zn is an essential trace element utilized in diverse cellular processes throughout the body, with well understood roles in both protein regulation and structure [124]. Zn is proposed as an intracellular second messenger (for reviews, see: [124,125]). Zn is a direct caspase cofactor in the regulation of the apoptotic cascade [126], and has been implicated in neurotransmitter regulation [127,128,129] and in cardiomyocyte function through the regulation of ryanodine receptor 2 (RyR2) [130,131]. A crucial cofactor, Zn is integral to the function of over 300 enzymes throughout all six classes (i.e., oxidoreductases, transferases, hydrolases, lyases, isomerases and ligases) [132]. The cellular concentration of Zn is tightly regulated by the orchestrated function of Zn-specific importers, exporters, and storage proteins [133].

Zn handling proteins are made up of two families: ZnT (SLC30) and ZIP (SLC39). The ZnT family functions to lower cytoplasmic Zn concentrations by transporting metal ions out of the intracellular space, acting as exporters. Inversely, the importing ZIP family functions to raise cytoplasmic Zn concentrations by trafficking metal ions into the intracellular space. MTs play a crucial role in the storage and buffering of Zn, with four distinct members of the family able to bind Zn ions (MT1, MT2, MT3, MT4) [134].

### 3.4. Toxic Copper and Zinc Deficiency

Systems biology approaches build evidence that Zn-dependent transcriptional regulation is likely impacted by excess Cu, though there is also evidence in WD mouse models that Zn-dependent enzymes are also impacted by excess Cu. These observations share intriguing similarities with a Zn-deficient mouse model where the Zn importer Zip14 is eliminated [135]. The Zip14 knockout (KO) (*Slc39A14*^-/-^) mouse has a phenotype of Zn deficiency that is mitigated by Zn supplementation in the diet [135]. Comparison of the *Atp7b*^-/-^ mouse (on C57Bl/6J background [136]) and the Zip14 KO mouse [135] reveals overlapping metabolite phenotypes with impacts on hepatic glucose metabolism and impaired gluconeogenesis in both models. Endosomes in Zip14 KO animals are Zn deficient with Zn-dependent insulin-degrading proteases, insulin-degrading enzymes and impaired cathepsin D, thus insulin receptor activity is increased. The *Atp7b*^-/-^ mouse has improved glucose tolerance and insulin sensitivity compared to wild type in a diet-induced obesity (DIO) model [136]. Cu excess in *Atp7b*^-/-^ animals promotes decreased steatosis, hepatic AMP-activated protein kinase (AMPK) activation, and decreased gluconeogenesis and lipogenesis with associated decreased levels of phosphoenolpyruvate carboxykinase-1 (PEPCK1), pyruvate carboxylase (PCX), fructose-bisphosphatase-1 (FBP1), fatty-acid synthase (FASN), acetyl-CoA carboxylase (ACC1) and SREBP-1c transcription. The mitochondrial form of phosphoenolpyruvate kinase, PEPCK2, and its mRNA levels increased in the *Atp7b*^-/-^ mouse. Zip14 KO mice also had decreased glucose, pyruvate, and lactate with the accumulation of TCA intermediates oxaloacetate, citrate, fumarate, and malate. These observations are also consistent with non-DIO *Atp7b*^-/-^: e.g., increased citrate [115] and decreased SREBP-1c transcripts [16].

Similarly, a transcriptomic study in a rat Zn deficiency model reveals impacted pathways also reported in WD mice. These include suppressed cholesterol synthesis genes (e.g., 3-hydro-3-methylglutaryl-CoA reductase) and NR transcripts (specifically PPAR-α) [117], suggesting that specific processes downregulated by Cu toxicity are also decreased in Zn deficiency.

The Zn-deficient mouse and rat models support the hypothesis that WD and Zn deficiency share some overlapping phenotypes with selective effects on metabolic processes. Zn deficiency is, however, rarely noted as a clinical feature of WD. However, one recent case of an early-onset WD patient with a concurrent mutation in MT1X reported Zn deficiency as the first presentation [137]. This patient had skin lesions similar to those in autosomal Zn deficiency acrodermatitis enteropathica (AE), related to mutation in the Zn transporter ZIP4 (SLC39A4). Oral Zn treatment improved symptoms in this patient, despite MT1X pathogenic mutation. This case supports a model that the mechanism of Zn treatment in Cu toxicity may be more complex than simply inducing an upregulation of MT.

It is possible that excess Cu, as illustrated in WD and WD models, promotes selective Zn deficiency. Cu–Zn interaction or competition in Cu toxicity is supported by other reports. Several studies find that specific Zn-dependent processes are disrupted by excess Cu. Increased hepatic Zn was reported in the young *tx* mouse [138], as was decreased abundance of carbonic anhydrase III (CA-III) [139], which are also phenotypes observed in the *Atp7b^-/-^* model [122]. In both models, hepatic Cu levels exceed hepatic Zn. Increased Zn and decreased CA-III initially appear inconsistent until one considers that complex formation between ligands and transition metals in cells follows the Irving–Williams Series [140], particularly when metals are in abundance and metal distribution is likely thermodynamically driven. In this case, Cu binding to ligand sites would be preferential to Zn binding.

Indirect readouts from metabolite analysis also find 2-fold increases in sorbitol and xylitol levels in the livers of *Atp7b*^-/-^ mice, along with a decrease in glyceraldehyde-3-phosphate [115]. In this case, xylitol would be synthesized from glucose via sorbitol and fructose [141]. This 2-fold change in downstream products would suggest a metabolic consequence resulting from a key processing enzyme (sorbitol dehydrogenase (SDH)) that is also Zn-dependent.

**Figure 1:**
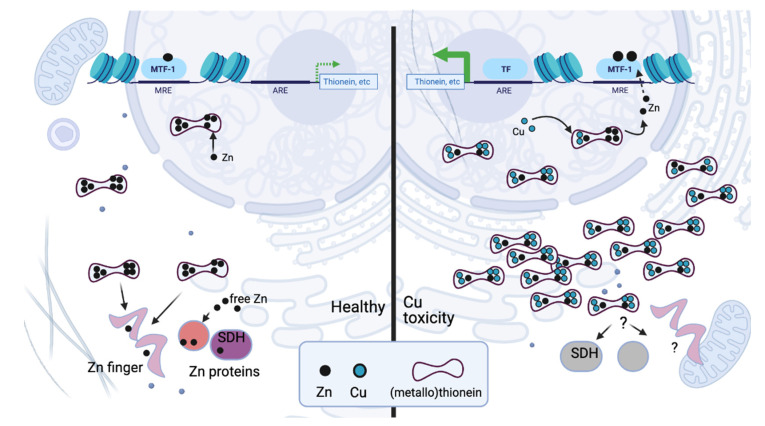
Proposed schematic of Cu interference with Zn distribution. A healthy cell is depicted on the left, with free Zn and Zn-metallothionein (MT) available as a labile Zn pool. A cell with Cu overload is depicted on the right, with increased MT binding available Zn as well as excess Cu. Cu in MT is in significant excess to Zn, interfering with distribution of the Zn pool. Figure created with BioRender: https://app.biorender.com/ (accessed on 19 March 2021).

Under normal (healthy) conditions, cells including hepatocytes typically contain 5–10-fold excess Zn over Cu. These metals will likely be bound to specific target proteins, resulting in kinetically-controlled Cu- and Zn-targeting through protein–protein interaction [142]. It is possible that in WD, Cu is in significant excess, and despite MT induction, Cu may be coordinated by other intracellular ligands such as glutathione. The result is that much Cu is available and able to compete with Zn for target sites (ligands). However, a targeted proteomics approach applied by Meacham et al. [122] indicated that in the six-weeks-old *Atp7b*^-/-^ mouse, the increase in MT1 and MT2 was sufficient to account for all of the additional Cu and Zn in *Atp7b*^-/-^ compared to wild type mouse liver. This work supports the model where Cu excess promotes increased MT1 and MT2 synthesis via MTF-1-induced transactivation. The increase in MTs would bind excess Cu but also available Zn. Thus, free Zn would be less available to proteins requiring it as a cofactor. Since some Zn-proteins including SDH and estrogen receptor (4-Cys NR) can acquire Zn from Zn-MT [143,144], these proteins would be more likely to encounter Cu in MT than Zn in MT, simply based on the ratio of Cu to Zn (illustrated in the schematic adapted from Meacham et al. [122]; Figure 1).

The above observations suggest that Cu toxicity may be mediated by Zn deficiency or selective impacts on Zn processes. It is notable that Zn deficiency has been associated with liver cirrhosis [145]; additionally, Zn deficiency may manifest as a spectrum of clinical features including cerebral and immune dysfunction, changes in taste and smell, loss of appetite, and impaired drug elimination. Furthermore, 10% of the human proteome includes a Zn cofactor [146]. The importance of Zn in cellular and metabolic processes cannot be overstated.

### 3.5. Copper and Zinc as Factors in Non-Wilson Pathology—Are They Linked?

Although Zn is an established treatment for WD that has low potential for toxicity, few studies have examined Zn-dependent systems as specific Cu targets in either animal models or WD patients. The observation that Zn and Cu interact or may have reciprocal regulation is not new. However, the most notable observations are where excess Zn consumption promotes Cu deficiency, including cases of induced Cu deficiency myeloneuropathy [147,148,149,150]. Examination of published work further illustrates the linkage between Cu and Zn in physiology.

Dietary Zn is shown to prevent Cu poisoning in sheep [151], supporting a competitive interaction between Cu and Zn and potential therapeutic benefit beyond the context of WD. Cu and Zn are linked as cofactors in Cu-Zn superoxide dismutase, where lack of Zn by the mutation of Zn coordination sites is cytotoxic to motor neurons [152]. Many of the liver associated diseases and Cu-responsive processes discussed above also implicate Zn imbalance or crosstalk between Cu and Zn. Zn deficiency is commonly reported in chronic liver diseases [145], while Cu deficiency is rarely reported, though possibly overlooked [153]. Cu accumulates as Cu-MT or Cu-Zn-MT in small (but not large) HCC tumors to a greater extent than in the surrounding non-tumor liver parenchyma [154]. Most illustrative, a retrospective study of 163 patients with cirrhosis reported that 83% were Zn deficient, with deficiency more prevalent in more severe disease and correlated with severity, infection and worse transplant-free survival [155]. Zn deficiency was also correlated with chronic liver disease progression in hepatitis virus-induced HCC [156]. These studies did not report Cu levels, so it is not clear whether Cu excess was associated with Zn deficiency in these diseases or whether both metals may be deficient. As discussed in Section 2.2, excess Cu has been reported in PBC and Cu chelation has been tested as a therapy [30,31,157]. Concurrently, bodily Zn may be deficient and oral Zn could be therapeutic in this disease by supplementing the Cu–Zn balance [158,159]. These observations are suggestive of Cu and Zn interaction in liver diseases, some of which are characterized by transition metal imbalance. Whether Cu and Zn levels are related in these diseases is still not clear.

## 4. Conclusions

WD patients as well as animal models of WD have provided substantial insight into Cu toxicity and potential therapies. The reducing environment in cells as well as detoxification mechanisms such as MTs likely mitigate much of the potential oxidative damage from Cu, but may additionally result in the disruption of Zn systems. It is plausible that oxidative damage observed with Cu toxicity is secondary to other specific Zn related metabolic consequences that are ultimately cytotoxic. Further research may extend these questions of Cu interaction with Zn machinery to other organ systems where Cu accumulation is observed, including studies in neurodegenerative Alzheimer’s Disease and Parkinson’s Disease.

## Figures and Tables

**Table 1:** Summary of Zn and chelator treatment studies in Wilson Disease.

Author, Year, Citation	Duration	Patient Count	Treatment	Short Term Outcomes (<1 Year)	Long Term Outcomes (>1 Year)
Beinhardt et al. 2014 [92]	14.8 years mean observation	229 (retrospective study)	D-PEN chelation therapy (dosage not reported)	N/A	35% stabilized, 24% improved, 26% recovered with chelation therapy, 15% deteriorated.
Brewer et al. 1998 [54]	12 years	141	Zn: variable between 3 × 50 mg/day and 1 × 25 mg/day	Reduction in urine, plasma and (minor) hepatic Cu. Increase in urine and plasma Zn. Partial improvement of neurological symptoms.	Urine Cu above normal. High urine and plasma Zn. Gradual reduction in non-CP plasma and liver Cu to normal values at years 8–12. Gradual neurological improvement over 6 years.
Brewer et al. 2001 [55]	5 years	34–4 (pediatric)	Zn: 50–150 mg/day depending on age of patient	Reduction in urine Cu and non-CP plasma Cu (*p* < 0.0001 and *p* < 0.05, resp). Increase in urine and plasma Zn (*p* < 0.0001, both). Speech measures improvement (*p* < 0.05). Neurologic measures improve (*p* < 0.05). Reduction in aminotransferases ALT, AST (*p* < 0.01)	Urine and non-CP plasma Cu stabilizing in normal ranges. Urine and plasma Zn stabilizing at high concentrations. Little long term (3 year) improvement in dysarthria. Continuing improvement of neurologic measures.
Bruha et al. 2011 [93]	15.1 years mean	117	Zn (17%); D-PEN (81%); 3 transplant (dosage not reported)	N/A	82% improvement in hepatic WD; 69% improvement in neurologic WD. Long-term survival similar to reference population.
Członkowska et al. 1996 [86]	12 years	67 (34-D-PEN, 33 Zn)	Zn: variable 600–800 mg/day	N/A	Similar improvements in patients between D-PEN and Zn treatment. Zn was better tolerated and had a greater rate of continuation through the 12 year period (88% Zn vs. 56% D-PEN)
Członkowska et al. 2014 [94]	5 years	143 (neurological: 35 D-PEN, 21 Zn; hepatic: 36 D-PEN, 51 Zn)	unknown	Similar frequency of improvement in neurological symptoms and liver enzymes.	Probability of not remaining on first-line therapy was higher for Zn than D-PEN in hepatic WD but similar in neurological WD. Adverse events more common with D-PEN than Zn (15% vs. 3%)
Dziezyc et al. 2014 [95]	Median 12 years (range 3–52)	87 (presymptomatic) 66.7% Zn treatment, 33.3% D-PEN	unknown	N/A	Positive treatment outcomes were similar between Zn and D-PEN with all patients. Non-compliant patients had significantly greater instances of neuro, hepatic and serum dysfunction or failure.
Farinati et al. 2003 [87]	12 years	67	Zn: 600–800 mg/day; D-PEN: 1–1.5 g/day	N/A	Of those that continued treatment through the period, 32% and 42% improved with D-PEN and Zn, respectively.
Haiman Hou et al. 2021 [83]	6 years	36	Zn: 2 × 25 mg/day in ages < 6 3 × 25 mg/day in ages 6–16 years 3 × 50 mg/day > 16 years	70% of patients had significant reductions in ALT with Zn monotherapy, 30% experienced treatment failure and added D-PEN	Patients improved to normal ALT levels with Zn monotherapy or Zn and D-PEN
Hoogenraad et al. 1987 [84]		27 9 patients Zn monotherapy, 8 patients developed D-PEN intolerance, 10 patients switched from D-PEN to Zn w/o developing intolerance	Zn: 3 × 200 mg/day in adults 3 × 100 mg/day in children	N/A	Eight of nine Zn patients had responded favorably to treatment, with a final patient dying in a hepatic coma. All eight patients with D-PEN intolerance improved with oral Zn, with two having deteriorating neurological symptoms during D-PEN treatment. Six of nine patients in the final group responded favorably, along with two asymptomatic patients.
Linn et al. 2009 [56]	24 years	17 7 hepatic patients, 5 neurologic patients and 5 with both	Zn: 136–276 mg/day	N/A	(median 12 years) Consistent and significant improvement in neurological patients (*p* < 0.01). No significant improvement in liver biochemistry in hepatic patients.
Marcellini et al. 2005 [81]	10 years	22 (pediatric)	Zn: 50–150 mg/day depending on age of patient	N/A	5 year: Reductions in AST, ALT and urinary Cu (*p* < 0.001) 10 year: No significant difference between 5- and 10-year outcomes. Significant reduction in Hepatic Cu (*p* = 0.001)
Merle et al. 2007 [96]	5 years	163 (retrospective)	Zn: 150–250 mg/day; D-PEN: 900–1800 mg/day; trientine: 900–2100 mg/day	N/A	76.1% improved or stable disease.
Svetel et al. 2009 [97]	15-years	142 (prospective)	Zn or D-PEN; (dosage not reported)		76.7% cumulative probability of survival, better prognosis with neurologic WD. Similar survival with Zn vs. D-PEN vs. combined.
Weiss et al. 2011 [85]	Median 17.1 years	288 (tertiary care centers, retrospective analysis)	Zn and D-PEN (dosage not reported)	N/A	Hepatic treatment failure more often in Zn monotherapy than with chelator or combination therapy. Zn treatment or chelators were effective in most patients; chelators were better at preventing hepatic deterioration.
Weiss et al. 2017 [98]	2 years	28 (prospective)	Bis-choline tetrathiomolybdate: 15–60 mg/day	N/A	71% met criteria for treatment success (25% decrease in non-ceruloplasmin-bound Cu). No drug-related neurological worsening. All stable liver function.
Wu et al. 2003 [80]	5 years	17 (presymptomatic)	Zn: 2 × 50 mg/day	No significant change in serum CP or urinary Cu	Significant reduction in Serum CP and urinary Cu at 5 years. No adverse effects in any Zn treated patients.

Abbreviations: Alanine transaminase (ALT), aspartate transaminase (AST), ceruloplasmin (CP), D-penicillamine (D-PEN).
